# Brain mechanisms of temporal processing in impulsivity: Relevance to attention-deficit hyperactivity disorder

**DOI:** 10.1177/23982128241272234

**Published:** 2024-08-13

**Authors:** Eleanor White, Jeffrey W. Dalley

**Affiliations:** 1 Department of Psychology, University of Cambridge, Cambridge, UK; 2 Department of Psychiatry, Herschel Smith Building for Brain and Mind Sciences, Cambridge, UK

**Keywords:** Subjective time estimation, pacemaker, striatum, prefrontal cortex, hippocampus, dopamine, stimulant drugs

## Abstract

In this article, we critique the hypothesis that different varieties of impulsivity, including impulsiveness present in attention-deficit hyperactivity disorder, encompass an accelerated perception of time. This conceptualisation provides insights into how individuals with attention-deficit hyperactivity disorder have the capacity to maximise cognitive capabilities by more closely aligning themselves with appropriate environmental contexts (e.g. fast paced tasks that prevent boredom). We discuss the evidence for altered time perception in attention-deficit hyperactivity disorder alongside putative underlying neurobiological substrates, including a distributed brain network mediating time perception over multiple timescales. In particular, we explore the importance of temporal representations across the brain for time perception and symptom manifestation in attention-deficit hyperactivity disorder, including a prominent role of the hippocampus and other temporal lobe regions. We also reflect on how abnormalities in the perception of time may be relevant for understanding the aetiology of attention-deficit hyperactivity disorder and mechanism of action of existing medications.

## Introduction

Attention-deficit hyperactivity disorder (ADHD) is a prevalent neurodevelopmental disorder associated with impaired sustained attention, impulsivity and hyperactivity (Drechsler et al., 2020). While it is typically diagnosed in children, with an estimated prevalence of roughly 5% (Polanczyk et al., 2007), longitudinal studies show that at least two-thirds of diagnosed youths retain symptoms into adulthood (Faraone et al., 2006). When combined with increasing awareness of the disorder, this may account for the increased diagnosis of ADHD in adults in recent years (Paris et al., 2015). However, despite this diagnostic shift, neuroimaging findings suggest a significant overlap in the neurobiological substrates of ADHD in children and adults (Cubillo et al., 2012). As a heterogeneous condition, ADHD is characterised by deficits in the diverse domains of inhibitory control, attention, reward processing and motor control (Faraone et al., 2015) and is frequently co-morbid with other psychiatric disorders, including substance use disorder (Charach et al., 2011). Nevertheless, as broadly acknowledged by others (Gonzalez-Carpio et al., 2017; Lawrence et al., 2008), and relevant to the concept of functional impulsivity (Dickman, 1990), the neurodivergent profile of individuals with ADHD can be advantageous in particular contexts; for example in situations where rapid decisions are needed for optimal outcomes.

A prominent idea first proposed by Barkley is that ADHD involves an inability to delay responding for future rewarded outcomes (Barkley, 1997). Thus, children with ADHD are posited to be *hyposensitive* to delayed rewards compared with unaffected children. Such individuals may even develop a ‘delay-averse motivational style’, which could also involve deficits in reward processing with delayed rewards being strongly devalued and thereby insufficient to reinforce behaviour. In contrast, others have suggested that deficits in executive functioning lie at the core of ADHD, including deficits in working memory and response inhibition (Lijffijt et al., 2005). While Sonuga-Barke has proposed a dual-pathway hypothesis, separating executive deficits and delay-aversion into distinct causal pathways for ADHD (Sonuga-Barke, 2003), these theoretical distinctions may not always be so clear-cut. Thus, delay-aversion has been shown to predict working memory performance (Karalunas and Huang-Pollock, 2011), suggestive of an interaction between delay-aversion and executive functioning, possibly mediated by existing deficits in executive functioning that exacerbate delay-aversion in ADHD.

An alternative theory of ADHD emphasises its temporal dimension whereby inter-individual differences in the perception of time are hypothesised to be responsible for variation in impulsive behaviour (Ptacek et al., 2019). Here time perception is defined as the rate at which cognitive functions reliant on temporal processing occur, in other words, their internal clock. Temporal perception is hypothesised to be consistently altered in those with ADHD causing subjective time to pass more quickly than real-world time, thereby producing a perception of time ‘dragging’. This disturbance in the perception of subjective time may explain overt symptoms of ADHD, such as heightened delay-discounting, as well as the context-dependency of behaviours present in ADHD (Delisle and Braun, 2011). Adding further nuance to the conceptualisation of ADHD, Waldren et al. (2024) recently reported that self-reported difficulties in attention do not map onto actual performance in an attentional control task. Thus, an altered perception of subjective time in ADHD and impulsive traits merits further analysis.

## Evidence for altered subjective time perception in ADHD

While events around us occur in real time, an individual interprets them with respect to their subjective time as a construction of the self (Wittmann, 2009). Evidence for anomalous subjective time in individuals with ADHD stems from several sources. For example, temporal deficits in the reproduction and estimation of set durations are a consistent finding in ADHD. Thus, when asked to estimate fixed durations of time children with ADHD tend to overestimate the interval, while underestimating similar durations when asked to reproduce them themselves (González-Garrido et al., 2008; Smith et al., 2002; Toplak et al., 2006). Such findings indicate that in ADHD subjective time passes more quickly than real time. In addition, children with ADHD are impaired on rhythmic finger-tapping tasks with a positive correlation found between impulsivity and the rate of finger tapping (Ben-Pazi et al., 2006) with associated impairments in timing signals in the motor system (Barratt et al., 1981).

Subjective time perception can be assessed experimentally by hyperbolic delay discounting procedures to model the influence of time perception on decision-making. Hyperbolic delay discounting describes the tendency to choose smaller immediate rewards over larger but delayed rewards (Ebert and Prelec, 2007) and is known to be elevated in ADHD (Jackson and MacKillop, 2016; McClure et al., 2014). A faster rate of subjective time in ADHD would essentially drive further reductions in the subjective value of delayed rewards by stretching the perceived temporal distance between present and future time points. Thus, an increased rate of subjective time passage traps individuals with ADHD in the present, leading to decisions which prioritise short-term outcomes over longer-term consequences (Paasche et al., 2019).

An increased rate of subjective time passage may also explain core symptoms of ADHD in children such as fidgeting and hyperactivity. Thus, individuals with ADHD would be expected to develop boredom at a faster rate, due to a demand for a greater level of environmental stimulation to remain alert. This may be the cause of children with ADHD turning towards alternative forms of stimulation, such as fidgeting or hyperactivity, theoretically to avoid the aversive state of boredom (McDougal et al., 2023). Such behaviour provides a distraction from the hyper-attentive state towards the passage of time that characterises boredom, especially in the context of under-stimulating environments. This interpretation of ADHD is supported by increases in the activity and inattention of hyperactive children during periods of delay (Antrop et al., 2002). The termination of boredom would thus be a key motivator for delay-minimisation in ADHD as supported by increased frustration following the occurrence of an unforeseen time delay (Sonuga-Barke, 1994). Consistent with this idea, a fixed trial length was shown to abolish differences in delay discounting between children with and without ADHD (Sonuga-Barke et al., 1998).

## Neural substrates of subjective time perception

**Figure 1.**
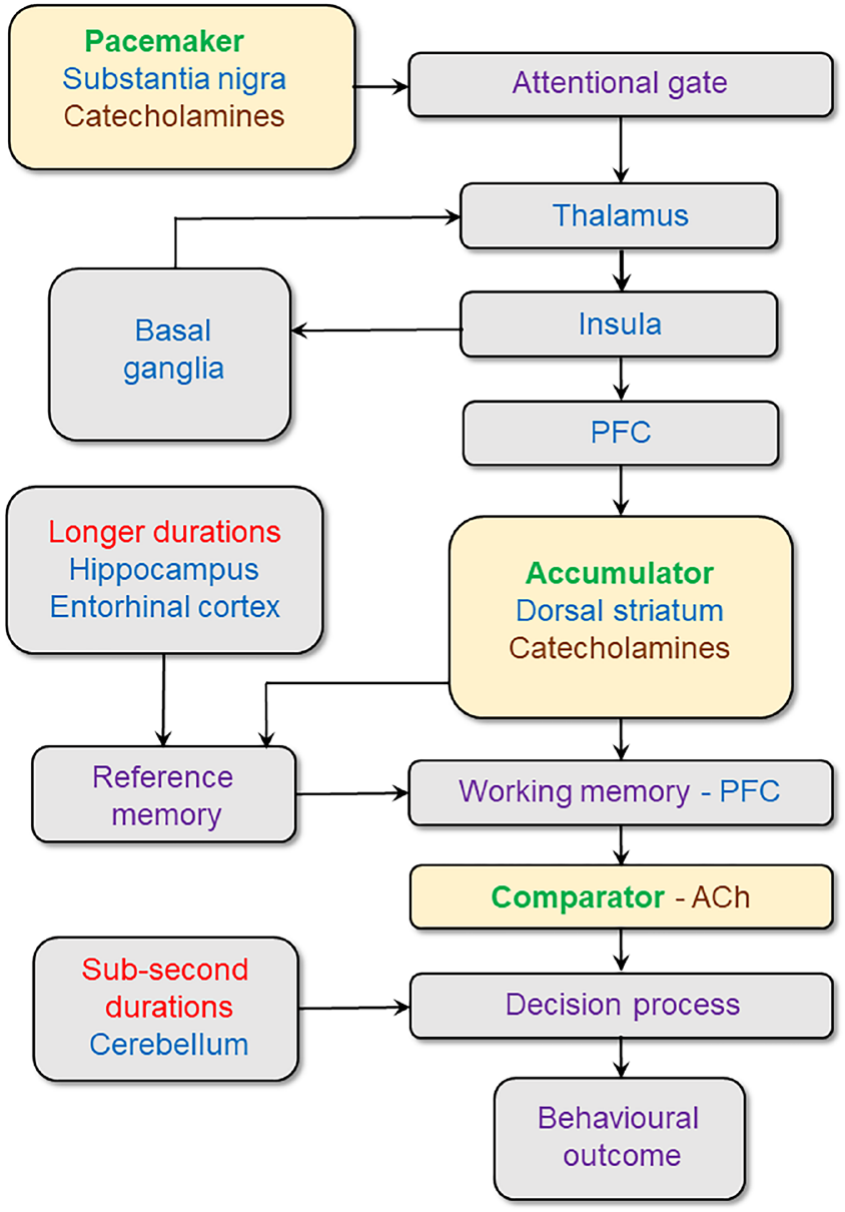
A schematic illustration of the putative neural network underlying the subjective perception of time. Candidate neural loci are based on the internal clock model (Treisman, 1963) with pacemaker, accumulator and comparator components, which are modulated by catecholaminergic (dopamine and noradrenaline) and cholinergic pathways. Deficits in some or all of the circuit’s nodes may dysregulate the temporal network to produce a quickened sense of subjective time estimation in individuals exhibiting impulsive characteristics.

We propose that subjective time passage can be explained by a neural mechanism which is itself the product of a distributed brain network involved in temporal processing. The activity of such a network depends on both the time duration perceived and a broader perceptual context and traditionally is consistent with the notion of an internal clock (Treisman, 1963). The internal clock describes a set of assigned timing elements for a particular task (i.e. pulse generators and accumulators), which are coordinated to produce the subjective percept of time. Such a model provides a useful starting point for identifying the neuroanatomical substrates of subjective time (see Figure 1). However, different scales of subjective time may also depend on different neural mechanisms and associated neuroanatomical substrates. For example, maintaining and recognising sub-second durations likely does not demand the same mnemonic resources as supra-second durations. Indeed, different neural structures may be involved in representing the distinction between sub- and supra-second durations (Coull et al., 2011; Matell et al., 2003). Moreover, recent conceptualisations of the neural basis of timing refer to models where time is represented by so-called population clocks that emerge from the dynamics of activity in distributed neural circuits (Paton and Buonomano, 2018). Such models putatively evoke clock-like processes that represent the forward passage of time as well as a reconstructive process for already-experienced intervals of time (Tsao et al., 2022). As discussed in the latter sections, this distinction may be relevant for how time is represented for different subtypes of impulsivity.

Figure 1 presents an orthodox framework for the anatomical connectivity of the brain’s temporal network, embedded within an internal clock model and modulated by specific neurotransmitter systems. Abnormalities in one or more nodes of the temporal networks may be responsible for a quickened perception of time in ADHD. It is important to note, however, that the proposed temporal network may act in an integrative manner within broader brain networks. For example, subjective time may be the product of interactions with circuitry involved in reward magnitude evaluation (Fung et al., 2021), salience (Sherman et al., 2021) and executive functioning (Witowska et al., 2020).

## Neural substrates of the pacemaker and accumulator in time perception

Based on earlier seminal research involving interval discrimination tasks and correlations between neural activity and timing performance, the brain loci of the pacemaker and accumulator were identified some years ago as the substantia nigra and dorsal striatum, respectively (Meck, 1996). The integrative functioning of the pacemaker and accumulator is thought to depend on an attentional gating mechanism (Block and Gruber, 2014) that determines the probability of pulse accumulation within corticostriatal-thalamocortical networks (Coull et al., 2004). Such loops, as shown in Figure 1, may work by modulating sensory inputs to the cortex *via* the thalamus, either through widening or narrowing the thalamic filter. Within this framework, the attentional gate thus exerts a tonic inhibitory effect on the accumulator. While such a mechanism may facilitate flexible modulation of subjective time in the neuro-normative population, constitutively altered reward processing by the striatum in ADHD (Furakawa et al., 2014) may be one route through which deficits in temporal perception arise.

As illustrated in Figure 1, the prefrontal cortex (PFC) may function as the neural substrate of the comparator, operating alongside the basal ganglia to compute subjective time from the number of pulses accumulated (Coull et al., 2004, 2011). This putative role requires working memory to hold information online about the number of pulses recorded and the ratio between pulses and duration over different time scales that exist in reference memory. In ADHD, the sense of time dragging could result from an increased rate of pulse production or accumulation or an altered ratio in reference memory of pulses to subjective. Such modifications could stem from impairments in working memory, hindering the ability to record pulses or to hold the reference memory pulse ratio ‘on-line’ for comparative purposes. Indeed, impaired working memory and other executive functions are acknowledged in ADHD (Nigg, 2005) and predict performance on measures of time perception (Lee and Yang, 2019).

Although incompletely specified, the role of the PFC as a comparator may be restricted to supra-second durations due to the greater importance of attentional resources over these timescales (Coull et al., 2011). Indeed, lesions and transcranial magnetic stimulation of the right PFC impair timing of supra, but not sub-second, durations (Jones et al., 2004). Furthermore, concurrent activation of the basal ganglia with the right PFC during duration estimation (Ferrandez et al., 2003) has led to the idea that recurrent loops forming a fronto-striatal circuit underlie the joint functions of the pacemaker-accumulator and comparator in subjective time. Notably, reduced activation of the right inferior PFC was detected in duration discrimination tasks in adolescents with ADHD (Rubia et al., 2009). Thus, deficits in the functioning of the PFC comparator could drive an altered perception of time in ADHD.

With regard to the so-called reference memory of temporal processing, it is unclear where this information is represented. One possibility is that reference memories referring to different timescales of durations are stored in specialised sub-systems. For example, the hippocampus may be involved in the reference memory for longer durations, such as minutes to hours, while the cerebellum may encode sub-second durations. Time-sensitive neurons, which respond to time intervals of around 2 min in durations, have been detected in the human hippocampus (Umbach et al., 2020) and may support episodic memory. In contrast, sub-second neural timing has been ascribed to the cerebellum (Ivry and Keele, 1989) and may underlie the encoding of precise temporal relationships important for motor learning and control. Thus, cerebellar and hippocampal systems may make specialised contributions to ‘reference memories’ over different durations. In line with this suggestion, the temporal deficits resulting from the abnormal function of these structures may have different consequences for behaviour. For example, deficits in sub-second subjective time may drive motor impulsivity, consistent with the role of the cerebellum in motor control and impairments in Go-No-Go tasks in ADHD (Trommer et al., 1988). This framework putatively suggests that hippocampal reference memories would feed directly to the fronto-striatal pacemaker-accumulator-comparator circuit when the need for mnemonic resources over longer periods was required. In contrast, the cerebellar system may operate over shorter timescales to contribute more immediately to behavioural control mechanisms.

## Neuromodulation of subjective time perception

Elucidation of the chemical neuromodulation of subjective time perception has putative relevance for understanding how amphetamine and other clinically efficacious drugs in ADHD produce their therapeutic effects. Using the curve-shift paradigm, whereby motivated responding and duration estimation are graphically represented on vertical and horizontal axes, it has been possible to dissociate modulatory effects of DA and acetylcholine (ACh) on pacemaker-accumulator processing and reference memory, respectively (Meck, 1996).

Catecholaminergic-targeting drugs have been reported to cause an immediate shift in the peak response time. For example, methamphetamine, a DA and noradrenaline (NA) reuptake inhibitor, shifts the peak earlier, while haloperidol, a D2 receptor antagonist, shifts the peak later (Meck, 1996). These results have been interpreted as an increase and decrease in the rate of pulse production, respectfully. Thus, as the rate of pulse production changes, comparisons with values stored in reference memory may act to drive alterations in subjective time perspective and thereby a corresponding shift in the peak response time. This effect may extend to temporal discounting in individuals with ADHD. Indeed, dysfunction of the left fronto-parieto-striato-thalamo-cerebellar network, seen during temporal discounting in ADHD, was abolished by methamphetamine (Rubia et al., 2009). In one noteworthy study, fibre photometry was used to record calcium activity in the substantia nigra during a task requiring mice to judge whether the interval between two sound stimuli was shorter or longer than 1.5 s (Soares et al., 2016). Here, mice were more likely to judge the duration as shorter during increased DA activity and longer when DA activity was low (Soares et al., 2016). In the same study, in vivo optogenetics was used to investigate the causality of DA neuronal activity in altering the subjective perception of time. The main findings showed that photo-activation of midbrain DA neurons slowed down the estimation of time while inhibition of this same population of neurons had the opposite effect. Importantly, there was no effect on reaction times suggesting that the DA manipulations affected processing within the temporal processing network rather than motivational or motor control mechanisms. It is important to note, however, that the effects of pharmacological activations of the brain DA systems on the subjective judgement of time are broadly opposite to the effects of pathway-selective activation of midbrain DA neurons (Soares et al., 2016). The reasons for these discrepant findings are unclear, but possible explanations include ancillary effects of systemically administered drugs on motivation and response control mechanisms, additional effects of psychostimulant drugs on the brain NA systems, and effects on different populations of DA neurons in the midbrain that unlike the Soares study would also extend to DA neurons in the ventral tegmental area.

In contrast to catecholamines, cholinergic drugs do not cause an immediate change in peak response time. Nonetheless, compounds that deplete or increase synaptic levels of ACh eventually do cause the peak response time to shift earlier or later, respectfully (Meck, 1996). These findings have been interpreted to suggest a role for PFC ACh in modulating the ratio between accumulated pulses and real-world time stored in reference memory (Rubia et al., 2009).

## Relationship between temporal representations and impulsivity subtypes

As discussed above, a reference memory for duration may form an integral input to the temporal network that underlies our perception of time. In this section, we consider the neural mechanisms of temporal representation, and the way in which the diversity of temporal representations may contribute to various impulsivity subtypes, including waiting impulsivity (Dalley and Ersche, 2018); premature responding and delay discounting (Dalley et al., 2011).

We propose that the way the brain represents space through multiple coding strategies (Hartley et al., 2014) may be mirrored in its representation of time. A functional advantage of this approach would be that different neural representations are better suited to different kinds of tasks, as has already been proposed in spatial information processing (Lisman, 2007). An obvious way that these temporal representations may differ would be in the timescales across which they exist. However, further classifications have been suggested to include the encoding of implicit *versus* explicit temporal information, with explicit temporal information being further divided into prospective or retrospective processing streams. Within this framework, Sawatani et al. (2023) argue that implicit temporal information involves ‘representations of the length of an event experienced’, whereas explicit temporal information refers to the ‘direct monitoring of the passage of time’. Furthermore, prospective representations are based on the current time elapsed while the estimation of retrospective time involves memory of experienced events.

This multifaceted representation of time in the brain mirrors the heterogeneity of impulsivity, as well as its heterogeneous manifestation in different subtypes of ADHD. Experimental measures of impulsivity have been important in demonstrating the fractionation of the impulsivity construct, with a broad division emerging between impairments in delayed gratification, as typically shown in delay discounting, and moment-to-moment decisions, as seen in heightened premature responding on continuous performance tasks (Dalley et al., 2011). While a multi-centre study of ADHD found that SSRT and delayed-discounting measures did not correlate significantly, deficits in each still defined the entire spectrum of ADHD disorder subtypes (Solanto et al., 2001), highlighting the importance of both to the neuropathology of ADHD. Further investigation of the neural substrates of these subtypes of impulsivity confirms their distinct, if sometimes overlapping, neural basis (Dalley et al., 2011). Plausibly, the contribution of different temporal representations to these distinct behavioural manifestations of impulsivity may underlie some of the differences in their neural circuitry.

## Temporal representations and premature responding

Responses that occur prematurely in anticipation of reward-predictive cues are a common manifestation of impulsive behaviour and can be assessed using various tasks in humans and rodents (Dalley and Robbins, 2017). In rodents, for example premature responses are readily measureable in the five-choice serial reaction time task (5CSRTT), showing wide individual variation in frequency, and well as tasks implementing differential reinforcement of low rates of responding (DRL) schedules. In the 5CSRTT, subjects are trained to detect visual targets to earn food rewards, with anticipatory premature responses signalled by reward omission and a brief timeout period, while in DRL schedules, subjects are trained to withhold from responding for generally longer periods. Temporal representations may thus direct behaviour in DRL to a greater extent. Despite the apparent role for the perception of time passing in premature responding, it is unlikely that this relies on exactly the same temporal representations involved in delay discounting. Compared with the explicit consideration of delay duration in delay discounting, the typically shorter timescales involved in premature responding may mean that implicit temporal representations are adequate to represent the length of an event experienced, and thus drive responding after the appropriate delay. Indeed, combined recordings of single unit activity in the mPFC and the ventral striatum of rats performing the 5CSRTT found that premature responses appear to result from a failure in the timing of the initiation of a waiting process prior to the onset of a target stimulus (Donnelly et al., 2015). Thus, delay discounting and premature responding may depend on different temporal representations with retrospective time estimations forming modulatory decision inputs in delay discounting while implicit representations of delay may act as a timer responsible for triggering the onset of waiting, and subsequent motor function, in premature responding.

The tendency for anticipatory premature responses is dramatically elevated by interventions that lower 5-HT neurotransmission. For example, intraventricular administration of a selective 5-HT neurotoxin resulted in increased premature responses in the 5-CSRTT (Harrison et al., 1997). In addition, optogenetic stimulation of 5-HT neurons in the dorsal raphe nucleus (DRN) has been shown to shift the preference of mice away from smaller, more immediate rewards, suggestive of a role of 5-HT DRN neurons in promoting waiting behaviour (Miyazaki et al., 2014). Supporting this idea, the activity of DRN 5-HT neurons diminished when a reward was delayed for longer than expected (Miyazaki et al., 2011). However, while the timing of this ramping activity is consistent with promoting waiting behaviour for the appropriate duration, it is unclear whether this neural activity encodes a temporal representation of the length of the expected waiting period, or whether the 5-HT neurons themselves are downstream of primary temporal representations which modulate the 5-HT neuronal ramping activity. While there is more work to be done to elucidate the precise contribution of 5-HT neurons to temporal representations over short timescales, their role in temporally-driven behaviours may be relevant for certain forms of impulsivity.

The medial entorhinal cortex (mEC) is a possible substrate for the temporal representations which may exist upstream of 5-HT ramping activity during waiting behaviour. The identification of time cells in the mEC which are similar to those found in the hippocampus (Kraus et al., 2015) is indicative of the encoding of implicit time. Furthermore, calcium imaging techniques used alongside virtual reality-generated linear tracks found that the mEC generates time-dependent sequences of neural activity during active waiting (Heys et al., 2020). This temporal representation could form the basis of a trigger mechanism for initiating waiting activity in the mPFC and striatum, and determining how long the waiting behaviour is maintained by serotonergic neurons. Furthermore, there is evidence for the importance of these mEC temporal representations in both the learning of task-specific waiting behaviour, as well as the discrimination of waiting durations. Optogenetic inactivation during the learning phase of a 6 s waiting time prevented mice from successfully learning to wait for food reward (Heys et al., 2020). Moreover, while inactivation during the post-learning waiting behaviour had no effect, neurotoxic lesions of the mEC significantly impaired discrimination performance during 20s trials (Vo et al., 2021). These functional differences highlight the importance of timescales in the behavioural relevance of temporal representations, even between durations of seemingly similar scales. While the temporal function of the mEC has previously been neglected for an almost exclusively spatial focus, these more recent articles have brought our attention to its role in waiting behaviour, and the application of temporal representations to interval timing. On this basis, deficits in these representations may form the basis of shortened latencies to waiting activity in the mPFC and striatum and/or dysfunctional serotonergic ramping activity.

## Temporal representations and delay discounting

Delay discounting is a classic choice-based procedure to assess the preference of individuals for immediately available rewards over larger but delayed rewards. This has been experimentally verified as a model of subjective time, characterised mathematically as a hyperbolic decrease in the tendency to choose the larger delayed reward when delays are progressively increased (Ebert and Prelec, 2007). The resulting logarithmic perspective of time, inferred from hyperbolic delay discounting, is steepened relative to ‘choice impulsivity’ (McClure et al., 2014), with higher rates of discounting present in ADHD (Luman et al., 2010). This suggests the abnormal stretching of the perceived temporal distance between the present and future time points, implicating internal temporal representations, which plausibly form decision inputs to these choices as the basis for exacerbated delay discounting in impulsive individuals.

When considering the temporal representations which may be involved in delay discounting, it is useful to consider the demands of these temporally oriented decisions. Decisions involving delay discounting may rely on a number of temporal representations coming together to facilitate the choice at hand. While more difficult to replicate in animals, human versions of this paradigm involve individuals making decisions over extended durations of days to weeks (Kirby and Petry, 2004). The longer timescales involved here compared with premature responding suggest the need for a different approach to temporal representations of such durations to effectively simulate the delay discounting decision-making process. The combined activity of different temporal representations in different neural substrates may be necessary for the development and storage of a ‘library of durations’, based on an individual’s personal experience of durations across their lifetime. Below, we discuss the distinct roles the hippocampus, lateral entorhinal cortex (lEC) and mPFC may play in this approach to temporal representations over longer timescales. This could allow common representations of duration to be coded non-linearly in the brain and then drawn on when needed to make a decision involving the comparison of delays of different duration across these longer timescales.

Furthermore, consideration of the effect of each delay duration on the relative value of a reward demands the explicit representation of each duration, as seen in explicit retrospective time estimation. It is currently unclear whether neural encoding of explicit temporal representations exists independently of implicit neural representations, or whether implicit encoding forms the upstream neural foundation of explicit representations. Indeed, there is some evidence that such implicit temporal representations support the formation of duration memory which is necessary for retrospective time estimation, suggesting that the latter may be true (MacDonald et al., 2011).

As mentioned above, retrospective time estimation depends on the memory of experienced durations. The implicit encoding of event duration by both the hippocampus and lEC are hypothesised to contribute to the formation of duration memory necessary for retrospective time estimation. Neurons that fire sequentially throughout the duration of an event have been detected in implicit timing tasks in the hippocampus (Pastalkova et al., 2008). Time-sensitive neurons have been shown to represent the passage of time during immobility, as well as while running at various distances on a variable speed treadmill (Kraus et al., 2015), thus distinguishing them from hippocampal place cells. Furthermore, as the duration of events increased, the time field of each neuron displaying sequential activity also increased reflecting their representation of relative time as opposed to absolute time (Kraus et al., 2013). Moreover, in a task involving two distinct forced treadmill running durations, groups of neurons in CA1 represented the two durations relative to each other (Shimbo et al., 2021). Thus, the neural activity of the hippocampus may form a gradient of temporal representations for different event durations.

In addition to temporal representations in the hippocampus, lEC neurons are reported to exhibit firing activity representative of event durations. In contrast to temporal coding in the hippocampus, the lEC appears to represent event durations through the ramping activity of single neurons, with some increasing or decreasing activity towards the end of a trial and others peaking at the middle or at the beginning and end of a trial (Tsao et al., 2018). While this differs from the hippocampus, both regions contain information on the relative duration of an event with the lEC providing additional information on the durations between events. This latter function may be of relevance to the formation of an integrated episodic memory in the hippocampus (Tsao et al., 2018). Notably, the lEC has only been found to contribute to implicit time perception and episodic memory on a several-minute scale, suggestive of the relevance of these temporal representations to delay discounting, which normally involves temporal estimation over supra-minute durations. The precise relationship between the temporal representations of the hippocampus and entorhinal cortex is currently unknown, although their extensive interconnectivity has led to the suggestion that the entorhinal cortex may contribute to the formation of hippocampal time cells (Eichenbaum, 2017).

Such neural representations may be expected to facilitate the comparison of the effects of different delay durations in delay discounting. However, the contribution of the hippocampus to explicit timing decisions in relation to delay discounting decisions is controversial. Thus, whereas hippocampal time cells have mainly been detected in the processing of implicit temporal information for experienced events (Sawatani et al., 2023), lesions of the hippocampus generally have no effect on explicit timing behaviour (Dietrich and Allen, 1998; Jacobs et al., 2013) or where effects have been noted these appear to be relatively temporary (Meck, 1988; Meck et al., 1987). Based on previous research, medial temporal lobe representations may be of particular relevance to retrospective time estimation. Consistent with this view, patients with temporal lobe damage are unimpaired on explicit prospective timing tasks (Shaw and Aggleton, 1994) but show impairments on tasks that assess explicit retrospective timing (Melgire et al., 2005). Furthermore, extensive evidence implicates the role of the hippocampus in delay discounting. Rats with lesions of the hippocampus (Cheung and Cardinal, 2005; McHugh et al., 2008) and patients with hippocampal damage (Gupta et al., 2009) show increased delay discounting (i.e. show preference for immediate, small-magnitude rewards). In addition, elderly participants with impaired episodic memory, linked to hippocampal dysfunction, displayed increased delayed discounting (Lempert et al., 2020). At a mechanistic level, the deletion of NMDA receptors in the hippocampal CA1 region impaired delay-discounting behaviour and disrupted delay-dependent neural activity in this region (Masuda et al., 2020). Thus, NMDA receptors appear to be critical for the formation of temporal representations in the hippocampus and thereby the accurate encoding of delay-dependent behavioural choice.

So how are these explicit representations of duration combined and compressed into a library of durations which can be both stored and accessed as needed? As summarised in our proposed temporal network, reference memory refers to the encoding of the ratio between pulses and duration to form a representation of time passing. This information is then applied to the accumulation of pulses in any context for the perception of duration. While temporal representations in the medial temporal lobe can be seen as the encoding of the durations of one-off events, they do not address how such representations are consolidated to form a common representation of duration which can be applied to retrospective temporal tasks, thereby forming a reference memory. Analyses of temporal representations and functions of the mPFC, alongside the existence of extensive anatomical connectivity between the hippocampus and mPFC, suggest a joint role in establishing common representations of duration, as part of a wider role in encoding common features across events. Notably, the same mechanism has already been proposed for spatial information and the generalisation of knowledge more widely (Samborska et al., 2022), reinforcing the idea that the brain may deal with space and time in similar ways. Such interactions in the temporal domain would underlie the formation of a library of durations, thus allowing the feature of event duration to be compared across and between episodes, thereby facilitating retrospective time estimation and, by extension, delay discounting. Supporting this view, the mPFC has been shown to bias hippocampal encoding towards features that capture commonalities across events (Mack et al., 2016). Furthermore, mPFC-hippocampal interactions are enhanced when individuals experience new events which overlap with existing knowledge (Zeithamova et al., 2012). Of note, during this form of learning, a bidirectional flow of information occurs between the mPFC and hippocampus. Increases in the coordination of theta oscillations between the hippocampus and mPFC during decision-making and spatially guided and rewarded mnemonic processes confirm the functional relevance of connections between these two regions (Morici et al., 2022).

## Concluding remarks

This article reviews the evidence for a heightened perception of subjective time passage in ADHD. We discuss the components of a distributed temporal network involved in the subjective perception of time, in particular including the hippocampus, wider temporal lobe structures and PFC. Our analysis suggests that different manifestations of impulsivity may be commonly determined by disturbances in the way timing signals are encoded and subjectively interpreted relative to the passage of real time. Deficits in subjective time perception may, in turn, affect the behaviour of individuals with ADHD in a context-dependent manner, especially in less taxing and under-stimulating environments. Understanding the molecular and circuit-level mechanisms of temporal representations and how these interact with overlapping large-scale brain networks mediating perception, motivation, cognition and action may be fundamental to understanding the aetiology and treatment of complex neurodevelopmental disorders such as ADHD.

## References

[bibr1-23982128241272234] Antrop I Buysse A Roeyers H , et al (2002) Stimulation seeking and hyperactive behavior in children with ADHD: A re-analysis. Perceptual and Motor Skills 95(1): 71–90. 12365278 10.2466/pms.2002.95.1.71

[bibr2-23982128241272234] Barkley RA (1997) Behavioral inhibition, sustained attention, and executive functions: Constructing a unifying theory of ADHD. Psychological Bulletin 121(1): 65–94. 9000892 10.1037/0033-2909.121.1.65

[bibr3-23982128241272234] Barratt ES Patton J Olsson NG , et al (1981) Impulsivity and paced tapping. Journal of Motor Behavior 13(4): 286–300. 15215075 10.1080/00222895.1981.10735254

[bibr4-23982128241272234] Ben-Pazi H Shalev RS Gross-Tsur V , et al (2006) Age and medication effects on rhythmic responses in ADHD: Possible oscillatory mechanisms? Neuropsychologia 44(3): 412–416. 16083921 10.1016/j.neuropsychologia.2005.05.022

[bibr5-23982128241272234] Block RA Gruber RP (2014) Time perception, attention, and memory: A selective review. Acta Psychologia 149: 129–133. 10.1016/j.actpsy.2013.11.003 24365036

[bibr6-23982128241272234] Charach A Yeung E Climans T , et al (2011) Childhood attention-deficit/hyperactivity disorder and future substance use disorders: Comparative meta-analyses. Journal of the American Academy of Child and Adolescent Psychiatry 50(1): 9–21. 21156266 10.1016/j.jaac.2010.09.019

[bibr7-23982128241272234] Cheung THC Cardinal RN (2005) Hippocampal lesions facilitate instrumental learning with delayed reinforcement but induce impulsive choice in rats. BMC Neuroscience 6: 36. 15892889 10.1186/1471-2202-6-36 PMC1156904

[bibr8-23982128241272234] Coull JT Cheng RK Meck WH (2011) Neuroanatomical and neurochemical substrates of timing. Neuropsychopharmacology 36(1): 3–25. 20668434 10.1038/npp.2010.113 PMC3055517

[bibr9-23982128241272234] Coull JT Vidal F Nazarian B , et al (2004) Functional anatomy of the attentional modulation of time estimation. Science 303(5663): 1506–1508. 15001776 10.1126/science.1091573

[bibr10-23982128241272234] Cubillo A Halari R Smith A , et al (2012) A review of fronto-striatal and fronto-cortical brain abnormalities in children and adults with Attention Deficit Hyperactivity Disorder (ADHD) and new evidence for dysfunction in adults with ADHD during motivation and attention. Cortex 48(2): 194–215. 21575934 10.1016/j.cortex.2011.04.007

[bibr11-23982128241272234] Dalley JW Ersche KD (2018) Neural circuitry and mechanisms of waiting impulsivity: Relevance to addiction. Philosophical Transactions of the Royal Society B: Biological Sciences 374(1766): 20180145. 10.1098/rstb.2018.0145 PMC6335458 30966923

[bibr12-23982128241272234] Dalley JW Robbins TW (2017) Fractionating impulsivity: Neuropsychiatric implications. Nature Reviews Neuroscience 18(3): 158–171. 28209979 10.1038/nrn.2017.8

[bibr13-23982128241272234] Dalley JW Everitt BJ Robbins TW (2011) Impulsivity, compulsivity, and top-down cognitive control. Neuron 69(4): 680–694. 21338879 10.1016/j.neuron.2011.01.020

[bibr14-23982128241272234] Delisle J Braun CM (2011) A context for normalizing impulsiveness at work for adults with attention deficit/hyperactivity disorder (combined type). Archives of Clinical Neuropsychology 26(7): 602–613. 21653627 10.1093/arclin/acr043

[bibr15-23982128241272234] Dickman SJ (1990) Functional and dysfunctional impulsivity: Personality and cognitive correlates. Journal of Personality and Social Psychology 58(1): 95–102. 2308076 10.1037//0022-3514.58.1.95

[bibr16-23982128241272234] Dietrich A Allen JD (1998) Functional dissociation of the prefrontal cortex and the hippocampus in timing behavior. Behavioral Neuroscience 112(5): 1043–1047. 9829782 10.1037//0735-7044.112.5.1043

[bibr17-23982128241272234] Donnelly NA Paulsen O Robbins TW , et al (2015) Ramping single unit activity in the medial prefrontal cortex and ventral striatum reflects the onset of waiting but not imminent impulsive actions. European Journal of Neuroscience 41(12): 1524–1537. 25892211 10.1111/ejn.12895 PMC4529742

[bibr18-23982128241272234] Drechsler R Brem S Brandeis D , et al (2020) ADHD: Current concepts and treatments in children and adolescents. Neuropediatrics 51(5): 315–335. 32559806 10.1055/s-0040-1701658 PMC7508636

[bibr19-23982128241272234] Ebert JEJ Prelec D (2007) The fragility of time: Time-insensitivity and valuation of the near and far future. Management Science 53(9): 1423–1438.

[bibr20-23982128241272234] Eichenbaum H (2017) On the integration of space, time, and memory. Neuron 95(5): 1007–1018. 28858612 10.1016/j.neuron.2017.06.036 PMC5662113

[bibr21-23982128241272234] Faraone SV Asherson P Banaschewski T , et al (2015) Attention-deficit/hyperactivity disorder. Nature Reviews Disease Primers 1: 15020. 10.1038/nrdp.2015.20 27189265

[bibr22-23982128241272234] Faraone SV Biederman J Mick E (2006) The age-dependent decline of attention deficit hyperactivity disorder: A meta-analysis of follow-up studies. Psychological Medicine 36(2): 159–165. 16420712 10.1017/S003329170500471X

[bibr23-23982128241272234] Ferrandez AM Hugueville L Lehéricy S , et al (2003) Basal ganglia and supplementary motor area subtend duration perception: An fMRI study. Neuroimage 19(4): 1532–1544. 12948709 10.1016/s1053-8119(03)00159-9

[bibr24-23982128241272234] Fung BJ Sutlief E Hussain Shuler MG (2021) Dopamine and the interdependency of time perception and reward. Neuroscience and Biobehavioral Reviews 125: 380–391. 33652021 10.1016/j.neubiorev.2021.02.030 PMC9062982

[bibr25-23982128241272234] Furakawa E Tripp G Bado P (2014) Abnormal striatal BOLD responses to reward anticipation and reward delivery in ADHD 9. PLoS ONE 9(2): e89129. 10.1371/journal.pone.0089129 PMC3935853 24586543

[bibr26-23982128241272234] Gonzalez-Carpio G Serrano JP Nieto M (2017) Creativity in children with attention deficit hyperactivity disorder (ADHD). Psychology 8(3): 319–334.

[bibr27-23982128241272234] González-Garrido AA Gómez-Velázquez FR Zarabozo D , et al (2008) Time reproduction disturbances in ADHD children: An ERP study. The International Journal of Neuroscience 118(1): 119–135. 18041610 10.1080/00207450601042177

[bibr28-23982128241272234] Gupta R Duff MC Denburg NL , et al (2009) Declarative memory is critical for sustained advantageous complex decision-making. Neuropsychologia 47(7): 1686–1693. 19397863 10.1016/j.neuropsychologia.2009.02.007 PMC2697903

[bibr29-23982128241272234] Harrison AA Everitt BJ Robbins TW (1997) Doubly dissociable effects of median- and dorsal-raphé lesions on the performance of the five-choice serial reaction time test of attention in rats. Behavioural Brain Research 89(1–2): 135–149. 9475622 10.1016/s0166-4328(97)00053-3

[bibr30-23982128241272234] Hartley T Lever C Burgess N , et al (2014) Space in the brain: How the hippocampal formation supports spatial cognition. Philosophical Transactions of the Royal Society B: Biological Sciences 369(1635): 20120510. 10.1098/rstb.2012.0510 PMC3866435 24366125

[bibr31-23982128241272234] Heys JG Wu Z Mascaro ALA , et al (2020) Inactivation of the medial entorhinal cortex selectively disrupts learning of interval timing. Cell Reports 32(12): 108163. 32966784 10.1016/j.celrep.2020.108163 PMC8719477

[bibr32-23982128241272234] Ivry RB Keele SW (1989) Timing functions of the cerebellum. Journal of Cognitive Neuroscience 1(2): 136–152. 23968462 10.1162/jocn.1989.1.2.136

[bibr33-23982128241272234] Jackson JN MacKillop J (2016) Attention-deficit/hyperactivity disorder and monetary delay discounting: A meta-analysis of case-control studies. Biological Psychiatry 1(4): 316–325. 27722208 10.1016/j.bpsc.2016.01.007 PMC5049699

[bibr34-23982128241272234] Jacobs J Weidemann CT Miller JF , et al (2013) Direct recordings of grid-like neuronal activity in human spatial navigation. Nature Neuroscience 16(9): 1188–1190. 23912946 10.1038/nn.3466 PMC3767317

[bibr35-23982128241272234] Jones CR Rosenkranz K Rothwell JC , et al (2004) The right dorsolateral prefrontal cortex is essential in time reproduction: An investigation with repetitive transcranial magnetic stimulation. Experimental Brain Research 158(3): 366–372. 15365666 10.1007/s00221-004-1912-3

[bibr36-23982128241272234] Karalunas SL Huang-Pollock CL (2011) Examining relationships between executive functioning and delay aversion in attention deficit hyperactivity disorder. Journal of Clinical Child and Adolescent Psychology 40(6): 837–847. 22023275 10.1080/15374416.2011.614578 PMC4649931

[bibr37-23982128241272234] Kirby KN Petry NM (2004) Heroin and cocaine abusers have higher discount rates for delayed rewards than alcoholics or non-drug-using controls. Addiction 99(4): 461–471. 15049746 10.1111/j.1360-0443.2003.00669.x

[bibr38-23982128241272234] Kraus BJ Brandon MP Robinson RJ , et al (2015) During running in place, grid cells integrate elapsed time and distance run. Neuron 88(3): 578–589. 26539893 10.1016/j.neuron.2015.09.031 PMC4635558

[bibr39-23982128241272234] Kraus BJ Robinson RJ White JA , et al (2013) Hippocampal ‘time cells’: Time versus path integration. Neuron 78(6): 1090–1101. 23707613 10.1016/j.neuron.2013.04.015 PMC3913731

[bibr40-23982128241272234] Lawrence A Clark L Labuzetta JN , et al (2008) The innovative brain. Nature 456(7219): 168–169. 19005531 10.1038/456168a

[bibr41-23982128241272234] Lee HY Yang EL (2019) Exploring the effects of working memory on time perception in attention deficit hyperactivity disorder. Psychological Reports 122(1): 23–35. 29417882 10.1177/0033294118755674

[bibr42-23982128241272234] Lempert KM Mechanic-Hamilton DJ Xie L , et al (2020) Neural and behavioral correlates of episodic memory are associated with temporal discounting in older adults. Neuropsychologia 146(1): 107549. 32621907 10.1016/j.neuropsychologia.2020.107549 PMC7502478

[bibr43-23982128241272234] Lijffijt M Kenemans JL Verbaten MN , et al (2005) A meta-analytic review of stopping performance in attention-deficit/hyperactivity disorder: Deficient inhibitory motor control? Journal of Abnormal Psychology 114(2): 216–222. 15869352 10.1037/0021-843X.114.2.216

[bibr44-23982128241272234] Lisman JE (2007) Role of the dual entorhinal inputs to hippocampus: A hypothesis based on cue/action (non-self/self) couplets. In: HE Scharfman (ed.) Progress in Brain Research, the Dentate Gyrus: A Comprehensive Guide to Structure, Function, and Clinical Implications. Amsterdam: Elsevier, pp. 615–818. 10.1016/S0079-6123(07)63033-7 17765741

[bibr45-23982128241272234] Luman M Tripp G Scheres A (2010) Identifying the neurobiology of altered reinforcement sensitivity in ADHD: A review and research agenda. Neuroscience and Biobehavioral Reviews 34(5): 744–754. 19944715 10.1016/j.neubiorev.2009.11.021

[bibr46-23982128241272234] MacDonald CJ Lepage KQ Eden UT , et al (2011) Hippocampal ‘time cells’ bridge the gap in memory for discontiguous events. Neuron 71(4): 737–749. 21867888 10.1016/j.neuron.2011.07.012 PMC3163062

[bibr47-23982128241272234] Mack ML Love BC Preston AR (2016) Dynamic updating of hippocampal object representations reflects new conceptual knowledge. Proceedings of the National Academy of Sciences of the United States of America 113(36): 13203–13208. 27803320 10.1073/pnas.1614048113 PMC5135299

[bibr48-23982128241272234] Masuda A Sano C Zhang Q , et al (2020) The hippocampus encodes delay and value information during delay-discounting decision making. Elife 9: e52466. 10.7554/eLife.52466 PMC7051257 32077851

[bibr49-23982128241272234] Matell MS Meck WH Nicolelis MA (2003) Interval timing and the encoding of signal duration by ensembles of cortical and striatal neurons. Behavioral Neuroscience 117(4): 760–773. 12931961 10.1037/0735-7044.117.4.760

[bibr50-23982128241272234] McClure J Podos J Richardson HN (2014) Isolating the delay component of impulsive choice in adolescent rats. Frontiers in Integrative Neuroscience 8: 3. 24478644 10.3389/fnint.2014.00003 PMC3902300

[bibr51-23982128241272234] McDougal E Tai C Stewart TM , et al (2023) Understanding and supporting attention deficit hyperactivity disorder (ADHD) in the primary school classroom: Perspectives of children with ADHD and their teachers. Journal of Autism and Developmental Disorders 53(9): 3406–3421. 35776263 10.1007/s10803-022-05639-3 PMC10465390

[bibr52-23982128241272234] McHugh SB Campbell TG Taylor AM , et al (2008) A role for dorsal and ventral hippocampus in inter-temporal choice cost-benefit decision making. Behavioral Neuroscience 122(1): 1–8. 18298243 10.1037/0735-7044.122.1.1 PMC2671844

[bibr53-23982128241272234] Meck WH (1996) Neuropharmacology of timing and time perception. Cognitive Brain Research 3(3–4): 227–242. 8806025 10.1016/0926-6410(96)00009-2

[bibr54-23982128241272234] Meck WH (1988) Hippocampal function is required for feedback control of an internal clock’s criterion. Behavioral Neuroscience 102(1): 54–60. 3355658 10.1037//0735-7044.102.1.54

[bibr55-23982128241272234] Meck WH Church RM Wenk GL , et al (1987) Nucleus basalis magnocellularis and medial septal area lesions differentially impair temporal memory. The Journal of Neuroscience 7(11): 3505–3511. 3681402 10.1523/JNEUROSCI.07-11-03505.1987 PMC6569024

[bibr56-23982128241272234] Melgire M Ragot R Samson S , et al (2005) Auditory/visual duration bisection in patients with left or right medial-temporal lobe resection. Brain and Cognition 58(1): 119–124. 15878732 10.1016/j.bandc.2004.09.013

[bibr57-23982128241272234] Miyazaki K Miyazaki KW Doya K (2011) Activation of dorsal raphe serotonin neurons underlies waiting for delayed rewards. The Journal of Neuroscience 31(2): 469–479. 21228157 10.1523/JNEUROSCI.3714-10.2011 PMC6623450

[bibr58-23982128241272234] Miyazaki KW Miyazaki K Tanaka KF , et al (2014) Optogenetic activation of dorsal raphe serotonin neurons enhances patience for future rewards. Current Biology 24(17): 2033–2040. 25155504 10.1016/j.cub.2014.07.041

[bibr59-23982128241272234] Morici JF Weisstaub NV Zold CL (2022) Hippocampal-medial prefrontal cortex network dynamics predict performance during retrieval in a context-guided object memory task. Proceedings of the National Academy of Sciences of the United States of America 119(20): e2203024119. 10.1073/pnas.2203024119 PMC9171913 35561217

[bibr60-23982128241272234] Nigg JT (2005) Neuropsychologic theory and findings in attention-deficit/hyperactivity disorder: The state of the field and salient challenges for the coming decade. Biological Psychiatry 57(11): 1424–1435. 15950017 10.1016/j.biopsych.2004.11.011

[bibr61-23982128241272234] Paasche C Weibel S Wittmann M , et al (2019) Time perception and impulsivity: A proposed relationship in addictive disorders. Neuroscience and Biobehavioral Reviews 106(1): 182–201. 30529361 10.1016/j.neubiorev.2018.12.006

[bibr62-23982128241272234] Paris J Bhat V Thombs B (2015) Is adult attention-deficit hyperactivity disorder being over-diagnosed? The Canadian Journal of Psychiatry 60(7): 324–328. 26175391 10.1177/070674371506000705 PMC4500182

[bibr63-23982128241272234] Pastalkova E Itskov V Amarasingham A , et al (2008) Internally generated cell assembly sequences in the rat hippocampus. Science 321(5894): 1322–1327. 18772431 10.1126/science.1159775 PMC2570043

[bibr64-23982128241272234] Paton JJ Buonomano DV (2018) The neural basis of timing: Distributed mechanisms for diverse functions. Neuron 98(4): 687–705. 29772201 10.1016/j.neuron.2018.03.045 PMC5962026

[bibr65-23982128241272234] Polanczyk G de Lima MS Horta BL , et al (2007) The worldwide prevalence of ADHD: A systematic review and meta-regression analysis. The American Journal of Psychiatry 164(6): 942–948. 17541055 10.1176/ajp.2007.164.6.942

[bibr66-23982128241272234] Ptacek R Weissenberger S Braaten E , et al (2019) Clinical implications of the perception of time in attention deficit hyperactivity disorder (ADHD): A review. Medical Science Monitor 25: 3918–3924. 31129679 10.12659/MSM.914225 PMC6556068

[bibr67-23982128241272234] Rubia K Halari R Christakou A , et al (2009) Impulsiveness as a timing disturbance: Neurocognitive abnormalities in attention-deficit hyperactivity disorder during temporal processes and normalization with methylphenidate. Philosophical Transactions of the Royal Society B: Biological Sciences 364(1525): 1919–1931. 10.1098/rstb.2009.0014 PMC2685816 19487194

[bibr68-23982128241272234] Samborska V Butler JL Walton ME , et al (2022) Complementary task representations in hippocampus and prefrontal cortex for generalizing the structure of problems. Nature Neuroscience 25(10): 1314–1326. 36171429 10.1038/s41593-022-01149-8 PMC9534768

[bibr69-23982128241272234] Sawatani F Ide K Takahashi S (2023) The neural representation of time distributed across multiple brain regions differs between implicit and explicit time demands. Neurobiology of Learning and Memory 199(4): 107731. 36764645 10.1016/j.nlm.2023.107731

[bibr70-23982128241272234] Shaw C Aggleton JP (1994) The ability of amnesic subjects to estimate time intervals. Neuropsychologia 32(7): 857–873. 7936168 10.1016/0028-3932(94)90023-x

[bibr71-23982128241272234] Sherman MT Fountas Z Seth AK , et al (2021) Trial-by-trial predictions of subjective time from human brain activity. PLoS Computational Biology 18(7): e1010223. 10.1371/journal.pcbi.1010223 PMC9262235 35797365

[bibr72-23982128241272234] Shimbo A Izawa EI Fujisawa S (2021) Scalable representation of time in the hippocampus. Science Advances 7(6): eabd7013. 10.1126/sciadv.abd7013 PMC7857679 33536211

[bibr73-23982128241272234] Smith A Taylor E Rogers JW , et al (2002) Evidence for a pure time perception deficit in children with ADHD. Journal of Child Psychology and Psychiatry, and Allied Disciplines 43(4): 529–542. 12030598 10.1111/1469-7610.00043

[bibr74-23982128241272234] Soares S Atallah BV Paton JJ (2016) Midbrain dopamine neurons control judgement of time. Science 354(6317): 1273–1277. 27940870 10.1126/science.aah5234

[bibr75-23982128241272234] Solanto MV Abikoff H Sonuga-Barke E , et al (2001) The ecological validity of delay aversion and response inhibition as measures of impulsivity in AD/HD: A supplement to the NIMH multimodal treatment study of AD/HD. Journal of Abnormal Child Psychology 29(3): 215–228. 11411784 10.1023/a:1010329714819

[bibr76-23982128241272234] Sonuga-Barke EJS (1994) Annotation: On dysfunction and function in psychological theories of childhood disorder. Journal of Child Psychology and Psychiatry 35(5): 801–815. 7962242 10.1111/j.1469-7610.1994.tb02296.x

[bibr77-23982128241272234] Sonuga-Barke EJS (2003) The dual pathway model of AD/HD: An elaboration of neuro-developmental characteristics. Neuroscience and Biobehavioral Reviews 27(7): 593–604. 14624804 10.1016/j.neubiorev.2003.08.005

[bibr78-23982128241272234] Sonuga-Barke EJ Saxton T Hall M (1998) The role of interval underestimation in hyperactive children’s failure to suppress responses over time. Behavioural Brain Research 94(1): 45–50. 9708838 10.1016/s0166-4328(97)00168-x

[bibr79-23982128241272234] Toplak ME Dockstader C Tannock R (2006) Temporal information processing in ADHD: Findings to date and new methods. Journal of Neuroscience Methods 151(1): 15–29. 16378641 10.1016/j.jneumeth.2005.09.018

[bibr80-23982128241272234] Treisman M (1963) Temporal discrimination and the indifference interval: Implications for a model of the ‘internal clock’. Psychological Monographs 77(13): 1–31. 10.1037/h0093864 5877542

[bibr81-23982128241272234] Trommer BL Hoeppner JA Lorber R , et al (1988) The Go – No-Go paradigm in attention deficit disorder. Annals of Neurology 24(5): 610–614. 3202613 10.1002/ana.410240504

[bibr82-23982128241272234] Tsao A Sugar J Lu L , et al (2018) Integrating time from experience in the lateral entorhinal cortex. Nature 561(7721): 57–62. 30158699 10.1038/s41586-018-0459-6

[bibr83-23982128241272234] Tsao A Yousefzadeh SA Meck WH , et al (2022) The neural bases of timing of durations. Nature Reviews in Neuroscience 23(11): 646–665. 36097049 10.1038/s41583-022-00623-3

[bibr84-23982128241272234] Umbach G Kantak P Jacobs J , et al (2020) Time cells in the human hippocampus and entorhinal cortex support episodic memory. Proceedings of the National Academy of Sciences of the United States of America 117(45): 28463–28474. 33109718 10.1073/pnas.2013250117 PMC7668099

[bibr85-23982128241272234] Vo A Tabrizi NS Hunt T , et al (2021) Medial entorhinal cortex lesions produce delay-dependent disruptions in memory for elapsed time. Neurobiology of Learning and Memory 185(2): 107507. 34474155 10.1016/j.nlm.2021.107507

[bibr86-23982128241272234] Waldren LH Leung FYN Hargitai LD , et al (2024) Unpacking the overlap between Autism and ADHD in adults: A multi-method approach. Cortex 173(6): 120–137. 38387375 10.1016/j.cortex.2023.12.016

[bibr87-23982128241272234] Witowska J Zajenkowski M Wittmann M (2020) Integration of balanced time perspective and time perception: The role of executive control and neuroticism. Personality and Individual Differences 163: 110061.

[bibr88-23982128241272234] Wittmann M (2009) The inner experience of time. Philosophical Transactions of the Royal Society B: Biological Sciences 364(1525): 1955–1967. 10.1098/rstb.2009.0003 PMC2685813 19487197

[bibr89-23982128241272234] Zeithamova D Dominick AL Preston AR (2012) Hippocampal and ventral medial prefrontal activation during retrieval-mediated learning supports novel inference. Neuron 75(1): 168–179. 22794270 10.1016/j.neuron.2012.05.010 PMC3398403

